# Supplementary material for: Global hypo-methylation in a proportion of glioblastoma enriched for an astrocytic signature is associated with increased invasion and altered immune landscape
Source: eLife. 2022 Nov 22;11:e77335. doi: 10.7554/eLife.77335 (PMC9681209; doi:10.7554/eLife.77335)
Supplement: Figure 2—source data 1. [file elife-77335-fig2-data1.zip › Figure_2_source_data_1/Figure_2C/homerResults/motif55.similar.html]

motif55

## Information for motif55

A
C
G
T
C
T
A
G
A
C
G
T
G
T
A
C
A
C
G
T
C
T
G
A
C
A
G
T
C
G
T
A
A
C
G
T
G
T
A
C
  
Reverse Opposite:  

C
A
T
G
C
G
T
A
C
G
A
T
G
C
T
A
A
G
C
T
G
T
C
A
A
C
T
G
C
G
T
A
G
A
T
C
G
T
C
A
  

|  |  |
| --- | --- |
| p-value: | 1e-10 |
| log p-value: | -2.366e+01 |
| Information Content per bp: | 1.815 |
| Number of Target Sequences with motif | 14.0 |
| Percentage of Target Sequences with motif | 1.19% |
| Number of Background Sequences with motif | 5.6 |
| Percentage of Background Sequences with motif | 0.12% |
| Average Position of motif in Targets | 93.2 +/- 43.5bp |
| Average Position of motif in Background | 90.8 +/- 62.2bp |
| Strand Bias (log2 ratio + to - strand density) | 2.1 |
| Multiplicity (# of sites on avg that occur together) | 1.14 |
| Motif File: | file (matrix) reverse opposite |

### Similar de novo motifs found

|  |  |  |  |  |  |  |  |
| --- | --- | --- | --- | --- | --- | --- | --- |
| Rank | Match Score | Redundant Motif | P-value | log P-value | % of Targets | % of Background | Motif file |
| 1 | 0.668 | A G T C C G A T C T G A A C G T G C T A A C G T C G A T C A T G | 1e-8 | -20.463535 | 9.01% | 4.86% | motif file (matrix) |
| 2 | 0.705 | C T G A C G A T C G T A C G A T G T A C C G A T G C T A C G A T T C G A A C G T G T A C C G A T T G C A G C A T C G T A | 1e-8 | -18.654815 | 0.60% | 0.03% | motif file (matrix) |
| 3 | 0.686 | A C G T A G T C A C T G T C G A C G A T G T C A A G C T T A G C | 1e-6 | -14.825078 | 1.53% | 0.36% | motif file (matrix) |
| 4 | 0.777 | A T G C C G A T C G T A A G C T C G T A A C G T | 1e-4 | -10.603491 | 37.41% | 31.80% | motif file (matrix) |
